# Retinal imaging in pre‐eclamptic pregnancy: systematic review

**DOI:** 10.1002/uog.70162

**Published:** 2026-01-14

**Authors:** D. Kitmiridou, I. Mitrogiannis, M. Charakida, K. H. Nicolaides

**Affiliations:** ^1^ Harris Birthright Research Centre for Fetal Medicine Fetal Medicine Research Institute, King's College Hospital London UK; ^2^ Department of Women and Children's Health School of Life Course and Population Sciences, Faculty of Life Sciences and Medicine, King's College London London UK; ^3^ School of Biomedical Engineering and Imaging Sciences, King's College London London UK

**Keywords:** central retinal artery Doppler, choroidal thickness, optical coherence tomography, optical coherence tomography angiography, pre‐eclampsia, pregnancy, retinal fundus photography, retinal imaging

## Abstract

**Objective:**

To explore potential changes in retinal structures in pregnant women with pre‐eclampsia (PE).

**Methods:**

This was a systematic review of the literature on retinal assessment in pregnancies complicated by PE. PubMed, EMBASE via Ovid, The Cochrane Library and Scopus databases were searched in July 2025 using an *a‐priori‐*designed protocol for studies examining the retina of pregnant women with an established diagnosis of PE or those who developed PE following retinal assessment. Randomized controlled trials and prospective and retrospective cohort, case–control and population‐based studies were eligible. Risk of bias was assessed using the Risk Of Bias In Non‐randomized Studies of Interventions version 2 (ROBINS‐I V2) tool. Due to substantial heterogeneity observed between studies in the methods of retinal assessment, retinal parameters studied and gestational age at retinal examination, a meta‐analysis was not performed.

**Results:**

The electronic database search yielded 1544 results, of which 75 were eligible for full‐text review and 24 studies were included in the systematic review. The methods of retinal assessment utilized in the included studies were optical coherence tomography (OCT), optical coherence tomography angiography (OCTA), retinal fundus photography and central retinal artery Doppler. Each method examined a variety of retinal parameters. Studies using OCT reported that the choroidal thickness was decreased, increased or not significantly different in women with PE *vs* non‐PE controls. With regard to retinal thickness, retinal nerve fiber layer thickness, macular thickness, ganglion cell layer thickness, choroidal vessel density and total retinal volume, most studies reported similar values in PE and non‐PE groups. For OCTA parameters, including foveal avascular zone area, vessel density in the deep and superficial capillary plexuses and choriocapillaris blood flow, some studies reported reduced values in the PE group, while others reported no significant differences between groups. Retinal fundus photography was performed in two studies; one reported reduced arteriolar and venular equivalents in women with PE, and the other reported reduced arteriole‐to‐vein ratio in women with severe PE *vs* without PE. One study used Doppler to assess the central retinal artery and reported a higher resistance index in those with PE compared to non‐PE controls. According to the ROBINS‐I V2 tool, the risk of bias was moderate in eight studies and serious in 16 studies.

**Conclusions:**

Findings on the changes in retinal structure and function in pregnancies complicated by PE are inconclusive. The degree of change, and whether the changes precede the clinical onset of PE or are a result of hypertension and associated cardiovascular sequalae, is uncertain. There is an unmet need for large‐scale prospective studies to address this uncertainty. © 2026 The Author(s). *Ultrasound in Obstetrics & Gynecology* published by John Wiley & Sons Ltd on behalf of International Society of Ultrasound in Obstetrics and Gynecology.

## INTRODUCTION

Pre‐eclampsia (PE) is a major cause of maternal−fetal morbidity and mortality, affecting approximately 5% of pregnancies^1^. Women with PE are at an increased risk of future cardiovascular complications; they are twice as likely to develop ischemic heart disease and have a higher chance of cardiovascular mortality compared with women who were normotensive during pregnancy^2^, ^3^. This is likely a consequence of PE acting as a marker of chronic underlying susceptibility to future disease, rather than PE causing harm which eventually leads to clinically apparent maternal cardiovascular and metabolic sequelae^4^, ^5^.

Retinal assessment has been proposed as an accessible way to examine the systemic microcirculation, with the potential to predict the development of hypertension and identify individuals at higher cardiovascular risk^6^, ^7^. Narrowing of the retinal arterioles has been identified as a potential marker for developing hypertension, which is hypothesized to be a result of increased vascular resistance^8^. Changes in the small vessels of the retina are thought to precede gross vascular changes, thus allowing for earlier detection of individuals at risk^9^. Assessment of the retinal microvasculature can also provide information on cumulative changes, rather than a snapshot of the circulatory status at the time of assessment^10^. Optical coherence tomography (OCT) has been used to study the choroid, a vascular network that provides oxygen and nutrients to the retina. Outside of pregnancy, changes in choroidal thickness have been associated with cardiovascular disease^11^. In pregnancy, there is emerging evidence that various cardiovascular indices can be used for the prediction of PE^12^. However, there is no consensus within the currently available evidence on the retinal changes associated with PE^13^.

The aims of this study were to conduct a systematic review of the available literature regarding retinal assessment in pregnancies complicated by PE and to assess the feasibility of a meta‐analysis. We aimed to explore potential changes in retinal structures in pregnant women with PE and determine whether specific retinal parameters can serve as early biomarkers of PE.

## METHODS

This systematic review adhered to the Preferred Reporting Items for Systematic reviews and Meta‐Analyses (PRISMA) checklist^14^ and followed an *a‐priori*‐designed search protocol, which was registered prospectively on PROSPERO (ID: CRD420251010666).

### Data sources and search strategy

We performed an electronic search of PubMed, EMBASE via Ovid, The Cochrane Library and Scopus databases on 6 July 2025, using a combination of the following keywords and medical subject heading (MeSH) terms: ‘optical coherence tomography’, ‘retina’, ‘fundus photography’, ‘retinal imaging’, ‘retinal vasculature’, ‘retinal vessels’, ‘choroidal thickness’, ‘pre‐eclampsia’, ‘preeclampsia’, ‘pregnancy‐induced hypertension’ and ‘HELLP syndrome’. The reference lists of other systematic reviews and the selected studies were screened manually to identify additional eligible studies that were missed by the aforementioned search.

### Eligibility criteria and study selection

We included studies examining the retina of pregnant women with an established diagnosis of PE or those who developed PE following retinal assessment. Randomized controlled trials and prospective and retrospective cohort, case–control and population‐based studies were eligible for inclusion. We included studies utilizing any kind of non‐invasive retinal imaging, as well as those in which pregnant women without PE were used as controls. We excluded the following studies: review articles, case reports, case series, Letters to the Editor, conference abstracts, retracted articles and study protocols; studies not written in English; studies that did not specify the definition used for PE; studies including women with gestational or pre‐existing hypertension or cardiovascular disease; studies assessing retinal abnormalities rather than changes in retinal parameters; studies that did not define the gestational age range at retinal examination; and studies in which retinal assessment was performed only postpartum.

Study selection was performed independently by two reviewers (D.K., I.M.), who screened titles and abstracts to identify potentially eligible studies. Full‐text articles were then reviewed to select studies suitable for inclusion. Discrepancies were discussed and, if consensus could not be achieved between the two reviewers, a third senior reviewer (K.H.N.) was consulted.

### Data extraction

For each study, we populated a predefined data extraction form, which included the title, year and journal of publication, name of the first author, details of the study population (including whether non‐PE or non‐pregnant controls were also examined), number of participants, gestational age range at the time of retinal assessment, method of retinal assessment and retinal parameters studied.

We reported the mean ± SD or median (interquartile range) of the retinal parameters examined. Studies considered for inclusion were assessed carefully to avoid duplicates and overlapping samples. If overlapping samples were detected, the study with the largest sample size was selected for data extraction.

### Assessment of risk of bias

The risk of bias was assessed independently by two reviewers (D.K., I.M.) using the Risk Of Bias In Non‐randomized Studies of Interventions version 2 (ROBINS‐I V2) tool. We examined the risk of bias across seven domains: presence of and adjustment for confounding factors, classification of interventions, participant selection criteria, any deviations from the intended intervention, reporting and management of missing data, outcome measurement, and selection of the reported results. We combined the level of risk assigned to each of these domains (low, moderate, serious or critical) to calculate an overall risk of bias for each included study.

### Synthesis of results

Due to the substantial heterogeneity in the methods of retinal assessment, retinal parameters studied and gestational age at retinal examination observed between the studies included in this systematic review, a meta‐analysis was not performed.

## RESULTS

### Database search results

**Figure 1:**
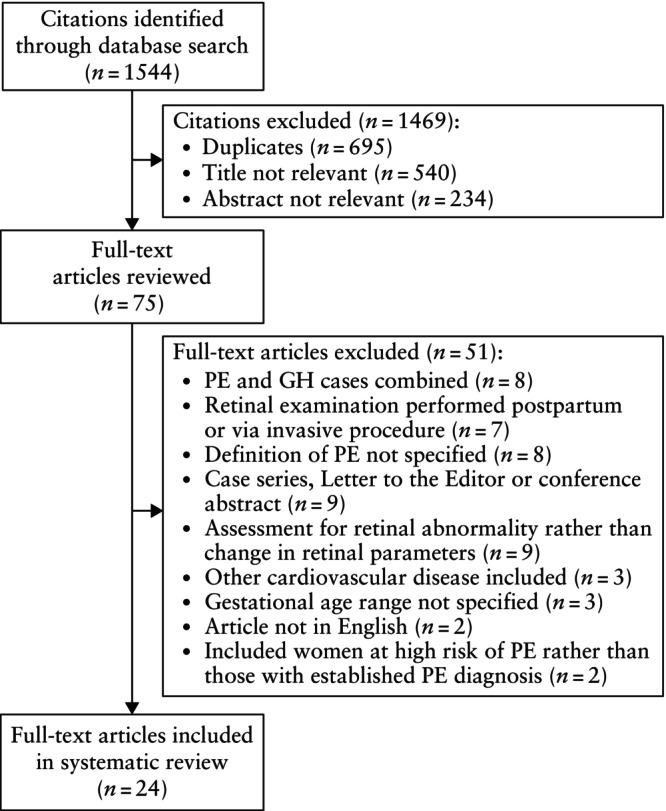
Flowchart summarizing inclusion of studies in systematic review, according to predefined criteria. GH, gestational hypertension; PE, pre‐eclampsia.

The electronic database search yielded 1544 results (Figure 1). Following the removal of duplicates, 849 citations underwent title and abstract screening, of which 774 were excluded. A total of 75 articles were eligible for full‐text review, of which 24 met the inclusion criteria and were ultimately included in the systematic review^15^, ^16^, ^17^, ^18^, ^19^, ^20^, ^21^, ^22^, ^23^, ^24^, ^25^, ^26^, ^27^, ^28^, ^29^, ^30^, ^31^, ^32^, ^33^, ^34^, ^35^, ^36^, ^37^, ^38^.

### Characteristics of included studies

Characteristics of the 24 included studies, including the number of PE and non‐PE cases, the method of retinal assessment, the gestational age range at retinal assessment and the retinal parameters examined, are presented in Table 1, ^15^, ^16^, ^17^, ^18^, ^19^, ^20^, ^21^, ^22^, ^23^, ^24^, ^25^, ^26^, ^27^, ^28^, ^29^, ^30^, ^31^, ^32^, ^33^, ^34^, ^35^, ^36^, ^37^, ^38^. The methods of retinal assessment used in the included studies were OCT, optical coherence tomography angiography (OCTA), retinal fundus photography and central retinal artery Doppler.

**Table 1:** Characteristics of 24 studies included in systematic review

Study	Women with PE (*n*)	Women without PE (*n*)	Retinal examination	GA at examination	Retinal parameter(s) examined
Dua (2025)^16^	42	42	OCT	32–37 w	Choroidal and retinal thickness
Naharwal (2025)^15^	30	30	OCT	20–39 w	Choroidal thickness
Erkan Pota (2024)^19^	27	30	OCTA/OCT	28–34 w	Choroidal, retinal, RNFL and GCL thickness; SCP, DCP and choriocapillaris vessel density; FAZ parameters
Lee (2024)^18^	66	0	OCT	26–41 w	Choroidal thickness and vascularity index
Tang (2024)^17^	64	63	OCTA/OCT	29–39 w	Retinal thickness; SCP vessel density; perfusion density; FAZ parameters
Fayed (2023)^21^	15	15	OCTA	Third trimester	Adjusted flow index
Kim (2023)^20^	46	7	OCT	32–36 w	Choroidal thickness and choroidal vessel density
Özcan (2022)^22^	50	50	OCTA/OCT	22 w to term	Macular and RNFL thickness; SCP and DCP vessel density; FAZ parameters; choriocapillaris blood flow area
Lee (2021)^23^	52	0	OCTA/OCT	21–39 w	Choroidal and GCL thickness; SCP and DCP vessel density; FAZ area
Sharudin (2020)^25^	50	50	OCT	28–41 w	Choroidal and macular thickness
Tok (2020)^24^	28	28	OCT	26–40 w	Macular, RNFL and GCL thickness
Benfica (2019)^29^	47	27	OCT	28–39 w	Choroidal thickness
Ciloglu (2019)^28^	55	43	OCTA/OCT	26–43 w	RNFL thickness; SCP and DCP vessel density; FAZ area
Evcimen (2019)^27^	52	54	OCT	20–39 w	Choroidal and macular thickness
Urfalıoglu (2019)^26^	27	26	OCTA	26–40 w	SCP and DCP vessel density; choriocapillaris and retinal blood flow area
Arab (2018)^30^	105	44	OCT	28–41 w	RNFL thickness
Duru (2016)^32^	32	41	OCT	28–37 w	Choroidal thickness
Kim (2016)^31^	7	14	OCT	28–37 w	Choroidal and retinal thickness
Ataş (2014)^34^	27	25	OCT	28–42 w	Choroidal, macular and RNFL thickness
Sayin (2014)^33^	33	46	OCT	16–36 w	Choroidal thickness
Lupton (2013)^35^	9	83	Fundus photography	13, 19, 29, 38 w	Central retinal arteriolar and venular equivalent
Gooding (2012)^37^	20	20	OCT	27–35 w	Retinal thickness and volume
Onaran (2012)^36^	20	21	Central retinal artery Doppler	24–38 w	PSV, EDV and RI of central retinal artery
Jaffe (1987)^38^	31	25	Fundus photography	Third trimester	Arteriole‐to‐vein ratio

Only first author is given for each study. DCP, deep capillary plexus; EDV, end‐diastolic velocity; FAZ, foveal avascular zone; GA, gestational age; GCL, ganglion cell layer; OCT, optical coherence tomography; OCTA, optical coherence tomography angiography; PE, pre‐eclampsia; PSV, peak systolic velocity; RI, resistance index; RNFL, retinal nerve fiber layer; SCP, superficial capillary plexus; w, weeks.

Grouping studies according to gestational age at retinal examination could not be performed accurately, because the original articles collected data over broad gestational age ranges, and the small study populations did not allow for meaningful comparisons between studies.

### Outcomes

#### Optical coherence tomography

**Figure 2:**
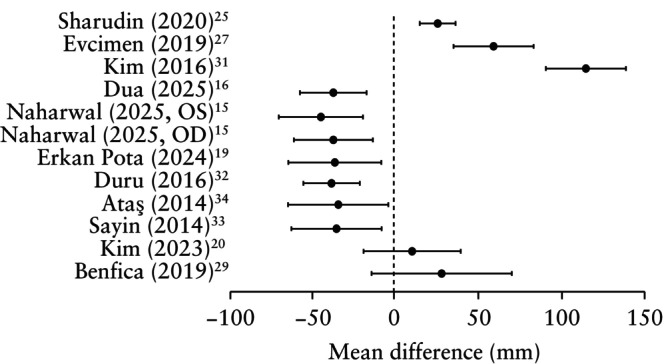
Forest plot of mean difference (95% CI) in choroidal thickness between women with *vs* those without pre‐eclampsia. Only first author is given for each study. OD, oculus dexter; OS, oculus sinister.

Choroidal thickness, assessed using OCT, was the most commonly examined retinal parameter, reported in 13 studies (Table 2). In women with PE, compared to those without PE, the choroid was thinner in six studies^15^, ^16^, ^19^, ^32^, ^33^, ^34^, thicker in three studies^25^, ^27^, ^31^ and not significantly different in two studies^20^, ^29^. The mean difference, with corresponding 95% CI, in choroidal thickness between women with and those without PE in each study is shown in Figure 2. Two studies did not include normotensive controls^18^, ^23^. One such study reported that, in women with PE, an increased soluble fms‐like tyrosine kinase‐1 to placental growth factor (sFlt‐1/PlGF) ratio was associated with a decrease in choroidal thickness^23^. In the other study, an increase in the urine protein‐to‐creatinine ratio was associated with an increase in choroidal thickness in women with PE^18^.

**Table 2:** Findings of studies utilizing optical coherence tomography to examine retinal parameters in pregnant women with pre‐eclampsia (PE)

Parameter/Study	Design	Finding
Choroidal thickness		
Sharudin (2020)^25^	PE *vs* no PE	Higher
Evcimen (2019)^27^	PE *vs* no PE	Higher
Kim (2016)^31^	PE *vs* no PE	Higher
Dua (2025)^16^	PE *vs* no PE	Lower
Naharwal (2025)^15^	PE *vs* no PE	Lower (OD); lower (OS)
Erkan Pota (2024)^19^	PE *vs* no PE	Lower
Duru (2016)^32^	PE *vs* no PE	Lower
Ataş (2014)^34^	PE *vs* no PE	Lower
Sayin (2014)^33^	PE *vs* no PE	Lower
Kim (2023)^20^	PE *vs* no PE	Similar
Benfica (2019)^29^	PE *vs* no PE	Similar
Lee (2024)^18^	PE	Associated directly with urine PCR
Lee (2021)^23^	PE	Associated inversely with sFlt‐1/PlGF ratio
Retinal thickness		
Dua (2025)^16^	PE *vs* no PE	Higher
Erkan Pota (2024)^19^	PE *vs* no PE	Similar
Tang (2024)^17^	PE *vs* no PE	Similar
Kim (2016)^31^	PE *vs* no PE	Similar
Gooding (2012)^37^	PE *vs* no PE	Similar
Retinal nerve fiber layer thickness		
Özcan (2022)^22^	PE *vs* no PE	Lower
Erkan Pota (2024)^19^	PE *vs* no PE	Similar
Tok (2020)^24^	PE *vs* no PE	Similar
Ciloglu (2019)^28^	PE *vs* no PE	Similar
Arab (2018)^30^	PE *vs* no PE	Similar
Ataş (2014)^34^	PE *vs* no PE	Similar
Macular thickness		
Özcan (2022)^22^	PE *vs* no PE	Lower
Sharudin (2020)^25^	PE *vs* no PE	Similar
Tok (2020)^24^	PE *vs* no PE	Similar
Evcimen (2019)^27^	PE *vs* no PE	Similar
Ataş (2014)^34^	PE *vs* no PE	Similar
Ganglion cell layer thickness		
Erkan Pota (2024)^19^	PE *vs* no PE	Similar
Tok (2020)^24^	PE *vs* no PE	Similar
Lee (2021)^23^	PE	Similar in women with high *vs* low sFlt‐1/PlGF ratio
Choroidal vessel density		
Kim (2023)^20^	PE *vs* no PE	Similar
Choroidal vascularity index		
Lee (2024)^18^	PE	Similar in women with high *vs* low PCR
Total retinal volume		
Gooding (2012)^37^	PE *vs* no PE	Similar

Only first author is given for each study. OD, oculus dexter; OS, oculus sinister; PCR, protein‐to‐creatinine ratio; sFlt‐1/PlGF ratio, soluble fms‐like tyrosine kinase‐1 to placental growth factor ratio.

When examining all other OCT parameters (retinal thickness, retinal nerve fiber layer thickness, macular thickness, ganglion cell layer thickness, choroidal vessel density and total retinal volume), the majority of studies did not find significant differences between women with and those without PE.

#### Optical coherence tomography angiography

Foveal avascular zone area was examined in five studies; measurements were similar in women with and those without PE^17^, ^19^, ^22^, ^28^, and among PE pregnancies, measurements were similar in women with high and those with low sFlt‐1/PlGF ratio^23^ (Table 3). Vessel density in the deep and superficial capillary plexuses was found to be either reduced or not significantly different in women with PE compared to normotensive controls^17^, ^19^, ^22^, ^26^, ^28^; no study demonstrated an increase in capillary plexus vessel density in women with PE. One study examined only women with PE and found that vessel density in the deep and superficial capillary plexuses was not associated with the sFlt‐1/PlGF ratio^23^.

**Table 3:** Findings of studies utilizing optical coherence tomography angiography and other imaging modalities to examine retinal parameters in pregnant women with pre‐eclampsia (PE)

Parameter/Study	Design	Finding
*Optical coherence tomography angiography*		
Foveal avascular zone area		
Tang (2024)^17^	PE *vs* no PE	Similar
Erkan Pota (2024)^19^	PE *vs* no PE	Similar
Özcan (2022)^22^	PE *vs* no PE	Similar
Ciloglu (2019)^28^	PE *vs* no PE	Similar
Lee (2021)^23^	PE	Similar in women with high *vs* low sFlt‐1/PlGF ratio
Deep capillary plexus vessel density		
Erkan Pota (2024)^19^	PE *vs* no PE	Lower
Deep foveal vessel density		
Özcan (2022)^22^	PE *vs* no PE	Lower
Ciloglu (2019)^28^	PE *vs* no PE	Similar
Deep parafoveal vessel density		
Ciloglu (2019)^28^	PE *vs* no PE	Similar
Urfalıoglu (2019)^26^	PE *vs* no PE	Similar
Deep perifoveal, parafoveal and foveal vessel density		
Lee (2021)^23^	PE	Similar in women with high *vs* low sFlt‐1/PlGF ratio
Superficial capillary plexus vessel density		
Erkan Pota (2024)^19^	PE *vs* no PE	Similar
Superficial foveal vessel density		
Özcan (2022)^22^	PE *vs* no PE	Lower
Ciloglu (2019)^28^	PE *vs* no PE	Similar
Superficial parafoveal vessel density		
Özcan (2022)^22^	PE *vs* no PE	Similar
Ciloglu (2019)^28^	PE *vs* no PE	Similar
Urfalıoglu (2019)^26^	PE *vs* no PE	Similar
Superficial perifoveal, parafoveal and foveal vessel density		
Tang (2024)^17^	PE *vs* no PE	Lower
Lee (2021)^23^	PE	Similar in women with high *vs* low sFlt‐1/PlGF ratio
Retinal perfusion density/blood flow area (perifoveal, parafoveal, foveal)		
Perfusion density		
Tang (2024)^17^	PE *vs* no PE	Lower
Retinal superficial blood flow area		
Urfalıoglu (2019)^26^	PE *vs* no PE	Similar
Retinal deep blood flow area		
Urfalıoglu (2019)^26^	PE *vs* no PE	Similar
Choriocapillaris blood flow		
Adjusted flow index		
Fayed (2023)^21^	PE *vs* no PE	Lower
Blood flow area		
Özcan (2022)^22^	PE *vs* no PE	Lower
Urfalıoglu (2019)^26^	PE *vs* no PE	Lower
Vessel density		
Erkan Pota (2024)^19^	PE *vs* no PE	Similar
*Retinal fundus photography*		
Corrected central retinal arteriolar equivalent		
Lupton (2013)^35^	PE *vs* no PE	Lower
Corrected central retinal venular equivalent		
Lupton (2013)^35^	PE *vs* no PE	Lower
Arteriole‐to‐vein ratio		
Jaffe (1987)^38^	sPE *vs* no PE	Lower
*Central retinal artery Doppler*		
Peak systolic velocity		
Onaran (2012)^36^	PE *vs* no PE	Similar
End‐diastolic velocity		
Onaran (2012)^36^	PE *vs* no PE	Similar
Resistance index		
Onaran (2012)^36^	PE *vs* no PE	Higher

Only first author is given for each study. PCR, urine protein‐to‐creatinine ratio; sFlt‐1/PlGF ratio, soluble fms‐like tyrosine kinase‐1 to placental growth factor ratio; sPE, severe pre‐eclampsia.

Measures of choriocapillaris blood flow were found to be lower in women with PE compared to normotensive controls in three out of four studies^19^, ^21^, ^22^, ^26^. Retinal perfusion density was lower in women with PE compared to those without PE in one study^17^, whereas in another study, retinal blood flow area did not differ significantly between groups^26^.

#### Other imaging modalities

In the two studies that used retinal fundus photography, retinal vascular changes were identified in women with PE^35^, ^38^ (Table 3). One study reported an overall reduction in corrected central retinal arteriolar and venular equivalents in women with PE compared to those without^35^. The other study reported a reduced arteriole‐to‐vein ratio in women with severe PE compared to normotensive controls, but there was no significant difference between cases with mild PE and those without PE^38^.

One study used Doppler to assess the central retinal artery and reported that there was a significantly higher resistance index, but similar peak systolic velocity and end‐diastolic velocity, in those with PE compared to normotensive controls^36^.

### Assessment of quality and risk of bias

The ROBINS‐I V2 tool was used to assess the risk of bias in the included studies (Table 4). In eight studies, there was an overall moderate risk of bias^16^, ^17^, ^18^, ^23^, ^24^, ^27^, ^33^, ^35^ and in the other 16, the overall risk of bias was serious^15^, ^19^, ^20^, ^21^, ^22^, ^25^, ^26^, ^28^, ^29^, ^30^, ^31^, ^32^, ^34^, ^36^, ^37^, ^38^.

**Table 4:** Risk‐of‐bias assessment of studies included in systematic review, using Risk Of Bias In Non‐randomized Studies of Interventions version 2 (ROBINS‐I V2) tool

Study	Confounding	Classification of intervention	Selection of participants	Deviation from intended intervention	Missing data	Measurement of outcome	Selection of reported results	Overall risk
Dua (2025)^16^	Moderate	Low	Low	Low	Low	Moderate	Low	Moderate
Naharwal (2025)^15^	Serious	Low	Moderate	Low	Low	Low	Low	Serious
Erkan Pota (2024)^19^	Serious	Low	Low	Low	Low	Moderate	Low	Serious
Lee (2024)^18^	Moderate	Low	Low	Low	Low	Moderate	Low	Moderate
Tang (2024)^17^	Moderate	Low	Low	Low	Low	Moderate	Low	Moderate
Fayed (2023)^21^	Serious	Low	Low	Low	Low	Low	Low	Serious
Kim (2023)^20^	Serious	Low	Moderate	Moderate	Moderate	Moderate	Low	Serious
Özcan (2022)^22^	Serious	Low	Low	Low	Low	Moderate	Low	Serious
Lee (2021)^23^	Low	Low	Low	Moderate	Low	Moderate	Low	Moderate
Sharudin (2020)^25^	Serious	Low	Low	Low	Low	Low	Low	Serious
Tok (2020)^24^	Moderate	Low	Low	Low	Low	Moderate	Low	Moderate
Benfica (2019)^29^	Serious	Low	Moderate	Low	Low	Low	Low	Serious
Ciloglu (2019)^28^	Serious	Low	Low	Low	Low	Low	Low	Serious
Evcimen (2019)^27^	Moderate	Low	Low	Low	Low	Low	Low	Moderate
Urfalıoglu (2019)^26^	Serious	Low	Moderate	Moderate	Low	Moderate	Low	Serious
Arab (2018)^30^	Serious	Low	Low	Low	Low	Moderate	Low	Serious
Duru (2016)^32^	Serious	Low	Moderate	Low	Low	Low	Low	Serious
Kim (2016)^31^	Serious	Low	Moderate	Low	Low	Moderate	Low	Serious
Ataş (2014)^34^	Serious	Low	Low	Low	Low	Low	Low	Serious
Sayin (2014)^33^	Low	Low	Moderate	Low	Low	Low	Low	Moderate
Lupton (2013)^35^	Moderate	Low	Low	Low	Low	Low	Low	Moderate
Gooding (2012)^37^	Serious	Low	Low	Low	Low	Moderate	Low	Serious
Onaran (2012)^36^	Serious	Low	Moderate	Low	Low	Moderate	Low	Serious
Jaffe (1987)^38^	Serious	Low	Low	Low	Low	Low	Low	Serious

Only first author is given for each study.

## DISCUSSION

### Main findings and interpretation

The studies included in this systematic review present conflicting findings regarding the effects of PE on the retina and its relevant structures. The discrepancies in choroidal thickness measurements likely reflect differences in disease severity, timing of assessment, imaging protocols and population characteristics. Thinning may indicate choroidal vasoconstriction and hypoperfusion, whereas thickening could represent inflammation, vascular leakage or impaired autoregulation^17^, ^39^, ^40^. Choroidal thinning was also associated with a higher sFlt‐1/PlGF ratio in women with PE in one study^23^, which could indicate progressive choroidal changes according to disease severity. Despite evidence of functional microvascular alterations in PE, other OCT‐derived parameters remained largely unchanged, suggesting that the gross retinal structure remains relatively preserved, at least in mild‐to‐moderate disease stages.

A higher level of agreement between studies was observed in the OCTA‐based findings. Measurements of choriocapillaris blood flow provided more consistent evidence of microvascular dysfunction, as it was found to be decreased in women with PE in three out of four studies^19^, ^21^, ^22^, ^26^. This reflects the lack of robust autoregulation of the choroidal vasculature, when compared with the retina, as it is a high‐flow, low‐resistance vascular bed and is highly sensitive to blood pressure alterations^41^. The possible reduction in vessel density in the superficial and deep capillary plexuses, along with decreased choriocapillaris perfusion, in women with PE likely reflects widespread endothelial dysfunction and impaired vascular autoregulation characteristic of PE pathophysiology.

Furthermore, foveal avascular zone parameters and retinal perfusion density metrics were unchanged in most studies^17^, ^22^, ^26^, ^28^, highlighting the need to determine which OCTA parameters are most sensitive to PE‐related changes. The lack of correlation between angiogenic markers (e.g. the sFlt‐1/PlGF ratio) and OCTA parameters reported by one study^23^ suggests that retinal microvascular changes may occur independently of biochemical profiles or may not be captured by current imaging thresholds.

Other modalities offered complementary insights. Retinal fundus photography, utilized by two studies^35^, ^38^, revealed reduced retinal vessel calibers in women with PE, particularly in severe cases, consistent with systemic vasoconstriction. A Doppler study of the central retinal artery identified a higher resistance index in women with PE^36^, supporting the notion of increased downstream vascular resistance, a common feature in hypertensive pregnancies.

It is important to highlight that different imaging modalities provide different information. OCT offers structural insights, whereas OCTA and retinal fundus photography help to visualize blood flow and the retinal vessels, respectively. Despite methodological heterogeneity, the emerging consensus suggests that PE is associated with subtle yet detectable alterations in the retinal microvasculature.

### Clinical perspectives

Retinal imaging is appealing in obstetric care due to its non‐invasive and repeatable nature. Zhou *et al*.^42^ demonstrated that fundus scores, generated from retinal fundus images obtained before 20 weeks' gestation, can be of value in predicting the subsequent development of PE. Wu *et al*.^43^ examined retinal vascular characteristics before 14 weeks as a biomarker for PE screening and predictor of adverse outcome when combined with maternal risk factors and mean arterial pressure. It is therefore possible that, when integrated with maternal cardiovascular assessment and uterine artery Doppler, which was surprisingly not attempted in any of the included studies, retinal imaging could enhance prediction models for PE and potentially guide personalized antenatal care. It is also worth exploring whether the addition of ophthalmic artery Doppler could further strengthen this approach. By detecting early hemodynamic changes, even before overt clinical symptoms of PE appear, ophthalmic artery Doppler could work synergistically with retinal imaging to provide a multimodal, multisystem screening strategy^44^, ^45^, ^46^, ^47^. Such a model, especially when combined with biomarkers like the sFlt‐1/PlGF ratio, could improve diagnostic precision, enable earlier intervention and reduce maternal−fetal morbidity^48^.

In addition, there is a large body of evidence examining retinal changes as a precursor of cardiovascular disease in non‐pregnant populations. Retinal imaging could, therefore, help identify those at highest risk of cardiovascular sequelae following the development of PE^1^, ^49^, ^50^.

Future research should aim to address key methodological limitations observed in the current literature. There is a need for prospective, longitudinal studies that assess retinal parameters at multiple timepoints throughout gestation and postpartum, to determine whether these changes precede, accompany or follow the clinical onset of PE. Large multicenter studies should incorporate well‐matched control groups and adjust for relevant confounders such as gestational age, pre‐existing hypertension, maternal body mass index and comorbidities. Finally, correlations with clinical outcomes, including maternal complications, fetal growth restriction and timing of delivery, will be crucial to establish the predictive value and clinical utility of retinal biomarkers.

### Strengths and limitations

Although there have been other systematic and narrative reviews on retinal assessment in pregnancies complicated by PE, this is the first to include studies regardless of the method of retinal assessment used and parameters examined, and to do so in a systematic manner. It highlights the variation in retinal imaging findings and possible pathophysiological explanations, ultimately drawing attention to the lack of consensus on the expected effect of PE on the retina.

A limitation of this review, and of the primary literature it encompasses, is the heterogeneity in study design, imaging protocols and population characteristics. Studies were varied in their definition and classification of PE, timing of imaging relative to disease onset, and selection of retinal parameters. Most studies were cross‐sectional, precluding assessment of causality or temporal progression. Lupton *et al*.^35^ were the only group that examined the retina in pregnant women from the first trimester onwards, with examinations performed at four different timepoints, and compared the findings between women who subsequently developed PE and those who remained normotensive throughout pregnancy. Their findings suggested that retinal arteriolar narrowing and an increase in peripheral vascular resistance may precede the clinical onset of PE. All other studies included in this systematic review examined women with established PE.

Furthermore, few studies adjusted for important confounders, such as gestational age, maternal body mass index, pre‐existing hypertension or diabetes mellitus — factors that may independently influence retinal vascular parameters^51^, ^52^. The small sample sizes and predominance of single‐center studies further limit generalizability and the potential for a meta‐analysis.

### Conclusions

Retinal imaging offers a promising, non‐invasive method for assessing microvascular health during pregnancy, and there is emerging evidence of its utility in identifying vascular dysfunction in PE. While findings remain inconsistent, key patterns support the biological plausibility of retinal involvement in PE.

Integration of retinal assessment into obstetric care, especially when combined with Doppler studies and angiogenic markers, could enable a multisystem screening strategy that supports early identification of PE, monitoring of disease progression and tailored intervention. Realizing this potential will require rigorous, standardized research to validate retinal biomarkers as meaningful, predictive tools in maternal health.

## Data Availability

Research data are not shared.
